# A Small Molecule Antagonist of PD-1/PD-L1 Interactions Acts as an Immune Checkpoint Inhibitor for NSCLC and Melanoma Immunotherapy

**DOI:** 10.3389/fimmu.2021.654463

**Published:** 2021-05-14

**Authors:** Yuanyuan Wang, Tingxuan Gu, Xueli Tian, Wenwen Li, Ran Zhao, Wenqian Yang, Quanli Gao, Tiepeng Li, Jung-Hyun Shim, Chengjuan Zhang, Kangdong Liu, Mee-Hyun Lee

**Affiliations:** ^1^ Department of Pathophysiology, School of Basic Medical Sciences, Zhengzhou University, Zhengzhou, China; ^2^ China-US (Henan) Hormel Cancer Institute, Zhengzhou, China; ^3^ Department of Immunology, The Affiliated Cancer Hospital of Zhengzhou University and Henan Cancer Hospital, Zhengzhou, China; ^4^ Department of Pharmacy, College of Pharmacy, Mokpo National University, Mokpo, South Korea; ^5^ Department of Pathology, The Affiliated Cancer Hospital of Zhengzhou University, Zhengzhou, China; ^6^ College of Korean Medicine, DongShin University, Naju, South Korea

**Keywords:** PD-1/PD-L1 inhibitor 1, PDI-1, PD-1/PD-L1, small molecule compound, immunotherapy, T cell activation

## Abstract

Immune checkpoint inhibitors, such as monoclonal antibodies targeting programmed death 1 (PD-1) and programmed death ligand-1 (PD-L1), have achieved enormous success in the treatment of several cancers. However, monoclonal antibodies are expensive to produce, have poor tumor penetration, and may induce autoimmune side effects, all of which limit their application. Here, we demonstrate that PDI-1 (also name PD1/PD-L1 inhibitor 1), a small molecule antagonist of PD-1/PD-L1 interactions, shows potent anti-tumor activity *in vitro* and *in vivo* and acts by relieving PD-1/PD-L1-induced T cell exhaustion. We show that PDI-1 binds with high affinity to purified human and mouse PD-1 and PD-L1 proteins and is a competitive inhibitor of human PD-1/PD-L1 binding *in vitro*. Incubation of *ex vivo* activated human T cells with PDI-1 enhanced their cytotoxicity towards human lung cancer and melanoma cells, and concomitantly increased the production of granzyme B, perforin, and inflammatory cytokines. Luciferase reporter assays showed that PDI-1 directly increases TCR-mediated activation of NFAT in a PD-1/PD-L1-dependent manner. In two syngeneic mouse tumor models, the intraperitoneal administration of PDI-1 reduced the growth of tumors derived from human PD-L1-transfected mouse lung cancer and melanoma cells; increased and decreased the abundance of tumor-infiltrating CD8+ and FoxP3+ CD4+ T cells, respectively; decreased the abundance of PD-L1-expressing tumor cells, and increased the production of inflammatory cytokines. The anti-tumor effect of PDI-1 *in vivo* was comparable to that of the anti-PD-L1 antibody atezolizumab. These results suggest that the small molecule inhibitors of PD-1/PD-L1 may be effective as an alternative or complementary immune checkpoint inhibitor to monoclonal antibodies.

## Introduction

In recent years, interest has been rekindled in immunotherapy for the treatment of cancer and other immune disorders (1). In particular, the introduction of immune checkpoint inhibitors (ICIs), which act by blocking negative regulatory pathways triggered by receptor–ligand interactions between tumor cells and T cells, has revolutionized the treatment of many cancers (2, 3). One example is the PD-1/PD-L1 immune checkpoint. PD-1 is an inhibitor of T cell proliferation and function (4) and plays a vital role in the physiological maintenance of immune tolerance as well as in tumor surveillance (5). PD-1 by its ligand PD-L1, expressed on antigen-presenting cells and tumor cells (6), induces negative signaling that counters signaling through the antigen-specific T cell receptor (TCR) and the costimulatory protein CD28 (7), leading to inhibition of T cell activation, proliferation, and cytokine production, and ultimately to apoptosis (8). Inhibition of PD-1/PD-L1 interactions therefore effectively rescues the activity of “exhausted” T cells and promotes their activation by antigen-expressing cells (9, 10). Accordingly, monoclonal antibodies (mAbs) that block the PD-1 or PD-L1 axis have shown remarkable benefits in clinical trials (11). Several mAbs have been approved by the US Food and Drug Administration for the treatment of various cancers, including nivolumab (anti-PD-1, Bristol Myers Squibb) for the treatment of melanoma (12) and non-small cell lung cancer [NSCLC (13)]; and atezolizumab (anti-PD-L1, Roche) for the treatment of triple-negative breast cancer (14), NSCLC (15). Anti-PD-1/PD-L1 mAbs have not only shown long-lasting beneficial responses in patients with a broad range of human cancers, but also displayed reduced toxicity compared with other ICIs, such as anti-CTLA-4 mAbs (16).

Small molecule compounds offer several advantages over mAbs as therapeutic agents, such as lower manufacturing costs, and less stringent storage conditions (17). Most importantly, the pharmacokinetics of small molecules are generally associated with good oral bioavailability, high tissue and tumor penetration, and long half-lives (18). Small molecule compounds can relatively easily traverse cellular membranes and other biological barriers, thus facilitating their access to intracellular targets. Finally, small molecules can be modified to ensure high efficacy and selectivity, and benign toxicity profiles. Therefore, small molecule drugs that target the PD-1/PD-L1 checkpoint could offer an attractive alternative and complementary therapies to existing mAbs (19).

In the present study, we performed computer modeling of the crystal structures of PD-1 and PD-L1 together with *in silico* screening to identify PDI-1 as a potential small molecule inhibitor of the PD-1/PD-L1 axis. To investigate the mechanism of action of PDI-1, we analyzed its binding to PD-1 and PD-L1 proteins *in vitro* and its ability to rescue TCR/CD28-dependent activation of T cells *ex vivo* and *in vivo* using melanoma and non-small-cell lung cancer (NSCLC) cell lines and mouse tumor models. We show that PDI-1 is a potent competitive inhibitor of PD-1/PD-L1 binding and suppresses tumor growth *in vivo* through a mechanism involving inhibition of TCR/CD28-dependent signaling, enhancement of anti-tumor cytotoxicity, and increased inflammatory cytokine production. Our results suggest that PDI-1 holds promise as a novel small molecule anti-cancer therapeutic agent.

## Materials and Methods

### Cell Culture

Lenti-X-293T (Human embryonic kidney cell line) were purchased from TaKaRa Bio, Inc. (Shiga, Japan). NCI-H1975, A549 (human non-small cell lung cancer), A375, SK-MEL-2 (human malignant melanoma)), and Jurkat (Human T-lymphocytes) cells were purchased from Cell Bank Australia (Shanghai, China), respectively. KLN205 (Murine lung cancer) and B16-F10 (Murine melanoma) cells were purchased from Cobioer bio (Nanjing, China). NCI-H1975, A549 and Jurkat cells were maintained in RPMI-1640 (Cat#01-100-1ACS, Biological Industries, Kibbutz Beit-Haemek, Israel) supplemented with 10% fetal bovine serum (FBS) (Cat#04-001-1ACS, Biological Industries) and 100U/ml penicillin-streptomycin (Cat#P1400, Solarbio, Beijing, China). A375, SK-MEL-2 and Lenti-X-293T cells were maintained in DMEM (Cat#01-052-1ACS, Biological Industries) supplemented with 10% FBS and 100 U/ml penicillin/streptomycin. B16-F10 was maintained in DMEM supplemented with 10% FBS and 100 U/ml penicillin/streptomycin. KLN205 was maintained in MEM (Biological Industries, Kibbutz Beit-Haemek, Israel) supplemented with 10% FBS, 100 U/ml penicillin/streptomycin, 0.1 mM non-essential amino acids (Cat#11140050, Gibco, Grand Island, NY, USA), 1 mM Sodium Pyruvate (Cat#11360070, Gibco, Grand Island, NY, USA).

### Isolation of Human PBMCs

PBMCs (Human peripheral blood mononuclear cells) were purchased from TPCS (Milestone Biotechnologies, Shanghai, China). CD3+ T cells were negatively selected from PBMCs by CD3 magnetic negative selection using EasySep Human T Cell Isolation Kit (Cat#17951, STEMCELL Technologies, Cologne, Germany) per manufacturer’s instructions. Human primary T cells were cultured in X-VIVO™15 medium (Cat#: BE02-060F, Lonza Group, Basel, Switzerland) with 5% FBS and 200 U/ml IL-2 (Cat#PHC0026, Thermo, Pudong, Shanghai, China). To activate T cells, a total of 3 million CD3+ T cells were treated by anti-CD3/CD28 magnetic Dynabeads (Cat#11161D, Thermo, Pudong, Shanghai, China) at the ratio of 1:1 together with 200 U/ml of IL-2, 50 ng/ml of IL-7 (Cat#PHC0075, Thermo, Pudong, Shanghai, China) and 50 ng/ml of IL-15 (Cat#PHC9151, Thermo, Pudong, Shanghai, China). After 48 h, activated CD3+ T cells were maintained by culture medium previously described at a density of 1 million cells per ml of culture medium and change the fresh medium every 2-3 days.

### Surface Plasmon Resonance (SPR) Assay

A BIACORE T200 (GE Healthcare, UK) was used to perform the SPR assay. Before starting the experiment, the machine was primed twice with ddH_2_O. Then a research-grade CM5 sensor chip was docked to the device and continued to prime twice with filtered phosphate-buffered saline (PBS). The PD-1 protein (Cat#10377-H08H, Sino Biological, Beijing, China) was immobilized using amine-coupling chemistry according to the wizard template. Briefly, the surface was first activated with a 1:1 mixture solution of 0.1 M NHS (N-hydroxy succinimide) and 0.1 M EDC (1-Ethyl-3-(3-dimethylaminopropyl)-carbodiimide hydrochloride) at 5 μl/min. Then a 20 ug/ml PD-1 protein, which was diluted in 10 mM sodium acetate (pH 4.5), was immobilized to channel 2 and then reached an expected density of 5000 RU. Channel 1 was left blank as a reference surface. Then channel 2 was blocked with an ethanolamine solution. To evaluate the binding ability, the PDI-1 (CAS#1675201-83-8, DC chemicals, Pudong, Shanghai, China) in PBS was injected over the two channels at a range that varies from 0.2 to 125 nM of PDI-1 concentration at a flow rate of 30 μl/min. The compound was designed for 90 and 300 s to associate and dissociated, respectively. The surfaces were renewed with a 5 s injection of 10 mM glycine (pH 2.5). PD-L1 protein (Cat#10084-H08H, Sino Biological, Beijing, China) was immobilized to channel 4 with the same method. The interaction of PD-L1 and PDI-1 was measured the same as above. The data were adjusted to a simple 1:1 interaction model within BiaEvaluation 3.1 software (Biacore, Uppsala, Sweden).

### Competitive ELISA Assay

To measure the inhibitory effects of PDI-1on interaction between PD-1 and PD-L1, we used the Acro Biosystems ELISA assay kit (Cat#EP-101, Acro Biosystems, Beijing, China) following the manufacturer’s instructions. Briefly, human PD-L1 were coated in a 96-well plate and incubated overnight at 4°C. Then, biotin-labeled human PD-1 protein was added, and incubation was performed for 20 min at room temperature. Next, the concentrations gradient of PDI-1 was added by dilution buffer, followed by incubation for 1 h at 37°C. After two washes followed by incubation with Streptavidin-HRP for 1h and TMB for 20 minutes at 37°C. Lastly, the absorbance at 450 nm was measured with Thermo Scientific Multiskan GO spectrophotometer (ThermoFisher Scientific, Vartaa, Finland).

### T Cell Cytotoxicity *In Vitro*

To validate the effect of primary T cells on cancer cells, we co-cultured primary T cell with cancer cells. H1975, A549, A375 or SK-MEL-2 cells were labeled by CFSE (carboxyfluorescein diacetate succinimidyl ester) (Cat# 65-0850-84, Invitrogene™ Bioscience, Shanghai, China) per manufacturer’s instructions, respectively. CFSE labeled H1975, A549, A375 or SK-MEL-2 cells (5 x 10^4^) were seeded into 12 well-plate overnight, then the next day incubated with primary T cells by the ratio of H1975, A549, A375 or SK-MEL-2 cells to T cells 1:5 for 18 h at the treatment of PDI-1. Next, co-culture cells were harvested, and then propidium iodide (PI) was stained 15 min on ice, last analyzed the sample using a BD FACSCalibur Flow Cytometer (BD Biosciences, San Jose, CA, USA).

To validate the effect of primary T cell on the cancer cells, we co-cultured primary T cell with hPD-L1-TCR-HEK-293T or TCR-HEK-293T cells labeled by CFSE per manufacturer’s instructions, respectively. CFSE labeled hPD-L1-TCR-HEK-293T or TCR-HEK-293T cells (5 x 10^4^) were seeded into 12 well-plate overnight, then the next day incubated with primary T cells by the ratio of hPD-L1-TCR-HEK-293T or TCR-HEK-293T cells to T cells 1:5 for 18 h at the treatment of PDI-1. Next, co-culture cells were harvested, and then propidium iodide (PI) was stained 15 min on ice, last analyzed the sample using a BD FACSCalibur Flow Cytometer (BD Biosciences, San Jose, CA, USA).

### hPD-L1 Expression of hPD-L1-TCR-HEK293T and TCR-HEK293T Cells

To validate the expression of hPD-L1 on hPD-L1-TCR-HEK293T and TCR-HEK293T cells, we incubated hPD-L1-TCR-HEK293T and TCR-HEK293T cells with PerCP/Cyanine5.5 anti-human PD-L1 antibody (Cat #329737, Biolegend) for 20 min on ice. Finally, we analyzed the sample using a BD FACSCalibur Flow Cytometer (BD Biosciences, San Jose, CA, USA).

### Construction of hPD-L1-TCR-HEK293T, TCR-HEK293T and hPD-1-NFAT-Jurkat Cells

hPD-L1-TCR-HEK293T and TCR-HEK293T: One day before transfection, seed HEK293T cells at a density of 2x10^6^ cells per ml, when cells reached 80% confluent at the time of transfection. The next day, transfect 1 μl TCR activator and hPD-L1 (Cat#79455, BPS Bioscience) or the only TCR activator (Cat#79455, Cat#79455) into cells following the manufacturer’s protocol. To sort the hPD-L1-TCR -HEK293T cells, we stain the cells with anti-human PD-L1 antibody (Cat #329706, Biolegend) by the FACSAria (BD) after 3 days transfection.

hPD-1-NFAT-Jurkat cells: Lentiviral packaging of the plasmid pLenti-NFAT-IRES-EGFP-PD-1 were performed by Gene Pharma. Lentivirus were infected into Jurkat cells at MOI 20 with 2µg/ml Polybrene, 7 days post infection, hPD-1-NFAT-Jurkat cells were sorted by FACSAria (BD), with staining of anti- human PD1 antibody (Cat #329906, Biolegend). Single clones were performed and selected by expression of PD-1.

### Fluorescent Multiplex Immunohistochemistry (mIHC)

The expression of murine CD8a, FoxP3 and PD-L1 were analyzed using Fluorescent multiplex immunohistochemistry. Tumor tissue sections were cut onto slides and heated at 60°C for 2h. Tumor tissue slides were then subjected to deparaffinization, rehydration and antigen retrieval, prior to endogenous peroxidase blocking. The slides were incubated with PD-L1(1:500, Cat#13684S, Cell Signaling Technology, Danvers, MA, USA), CD8a (1:100, Cat# 14-0081-86, eBioscience, Vienna, Austria) or FoxP3 (1:500, Cat# 14-5773-82, eBioscience, Vienna, Austria) primary antibody followed by the application of polymeric Horseradish peroxidase (HRP)-conjugated secondary antibodies ((Invitrogen™ eBioscience, Shanghai, China)). An appropriate fluorophore conjugated TSA (Invitrogen™ eBioscience, Shanghai, China) was then added at 1:100 dilution. The slides were rinsed with PBS after each step. Following TSA deposition, the slides were again subjected to antigen retrieval to strip the tissue-bound primary/secondary antibody complexes and ready for labeling of the next marker. These steps were repeated until all three markers were labeled and finally added with DAPI (Cat#P36935, Invitrogen™ eBioscience, Shanghai, China) at 1:20000 dilution. Then images were analyzed by Confocal microscopy at 60x magnification using the Nikon A1 laser confocal microscope (Nikon Instruments, Melville, NY, USA). For statistical analysis of grey intensities of fluorescent IHC signals, Image-Pro Plus 6.0 software (Media Cybernetics Inc, Maryland, USA) was used.

### Multiplex Cytokines Assay

The production in mice serum was measured by LEGEND plex™ Mouse Inflammation Panel. Blood was collected from the abdominal aorta of the mice after sacrifice and the serums were separated by centrifuge at 350g, 4°C for 15 min. Next, concentrations of IL-1α, IL-1β, IL-6, IL-10, IL-12p70, IL-17A, IL-23, IL-27, MCP-1, IFN-β, IFN-γ, TNF-α, and GM-CSF were measured using LEGEND plex™ Mouse Inflammation Panel (13-plex) (Cat#740446, Biolegend, Shenzhen, China). The samples were analyzed by FACS using FACSCalibur (BD). The data were analyzed *via* LEGENDplexv8.0.

### Single-Cell Dissociation From Tumor Tissues

The expression of CD3, CD4, CD8a in the TEM were measured by flow cytometry. Tumor tissues were harvested after injection and digested with Collagenase II (1 mg/ml, Sigma-Aldrich, C685, Pudong, Shanghai, China) in DMEM, which contained DNase (0.3mg/ml, Sigma-Aldrich, DN25) at 37°C with 800 rpm for 1 h. Afterward, the cell suspensions were filtered through a 70 µm strainer. Single-cell suspensions were stained with PerCP/Cyanine5.5 conjugated anti-mouse CD3 (Cat # 100217, clone 17A2, Biolegend), PE-conjugated anti-mouse CD8a (Cat #100707, clone 53-6.7, Biolegend), and APC conjugated anti-mouse CD4 (Cat #100411, clone GK15, Biolegend) for 30 min on ice, following the manufacturer’s instructions. Samples were analyzed by flow cytometry system and the data were analyzed by FlowJo software (Ashland, OR, USA).

### MRM

The PDI-1 was solved by ethanol to obtain the 2.6mg/ml working solution. Working standard solutions were diluted at 10ng/ml, 50ng/ml, 200ng/ml, 400ng/ml, 600ng/ml, 800ng/ml, 1000ng/ml, 1200ng/ml, 1400ng/ml concentration by ethanol. The serum calibration standards were obtained by adding working standard solutions into blank mice serum. A dose of 8mg/kg PD-I1 was administrated by i.p. to study the pharmacokinetic of PD-I1 in Balb/c male mice (5-week). Blood samples were collected at 1min, 5min, 10min, 20min, 0.5h, 1h, 2h, 4h, 8h, 16h, 24h time-point, and harvest the serum by centrifuging at 500g, 5 min. To extract PDI-1, serum was treated with ten volumes of ethanol and vortex. After centrifuging for 10 min at 10000 x g (4°C), the supernatant was dried in a SpeedVac, suspended in 50 μL of ethanol, and injected into the LC-MS/MS system. An Eclipse Plus C18 (100 × 2.1 mm; 3.5 µm) from Agilent (ZORBAX, USA) was used for chromatographic separation and isocratic elution consisted of water (A) and methanol (B), the ratio of A and B is 20:80. The injection volume was 5 μL and a flow rate of 0.3 ml/min was used. Concentration curves were used for quantitation of the peptides in the serum samples. The mass spectrometry analysis was processed using an Agilent Technologies 6460 Triple Quadrupole LC/MS (ZORBAX, USA).

### Mice

For this research, C57BL/6 and DBA/2 mice free of pathogens were purchased from Beijing Vital River Laboratory Animal Technology Co., Ltd (Beijing, China). Male and female mice between 4-5 weeks old were used for the syngeneic model. Mice were housed in a pathogen-free environment under conditions of 20°C ± 2°C, 50% ± 10% relative humidity, 12-h light/dark cycles. They were provided with food and water ad libitum. All animal experiments were generated with the institutional guidelines and approved by the Ethics Review Commission of Zhengzhou University (CUHCI2019001).

### Syngeneic Animal Experiments

PD-L1 humanized B16F10 and KLN205 (5 x 10^6^) cells were inoculated subcutaneously (s.c.) on the flank of mice with 100 μl of sterile PBS. Seven days following injection when the tumor volume reached 100 mm^3^, mice were randomized into three groups, and treatment was initiated. The vehicle or PDI-1 (2/4/8 mg/kg per mice) was administered intraperitoneally (i.p) every day. The tumor’s volume was measured twice a week using the formula (L * W * H * 0.52). When the average tumor volume of the vehicle group reached 1000 mm^3^, mice were sacrificed and the amount of liver, spleen and tumor were measured.

### Statistical Analysis

Data analysis was performed using GraphPad Prism7 (GraphPad Software Inc. La Jolla, CA, USA). Unpaired two-tailed Student’s t test was used to compare two groups; One way ANOVA followed by Bonferroni’s posttest was applied to assess the statistical significance of differences between multiple treatment groups. P values  ≤ 0.05 were considered statistically significant.

### Graphical Illustrations

Schematic illustrations were established with Bio Render (BioRender.com).

## Results

### PDI-1 as a Small Molecule Inhibitor of PD-1/PD-L1 Binding

**Figure 1:**
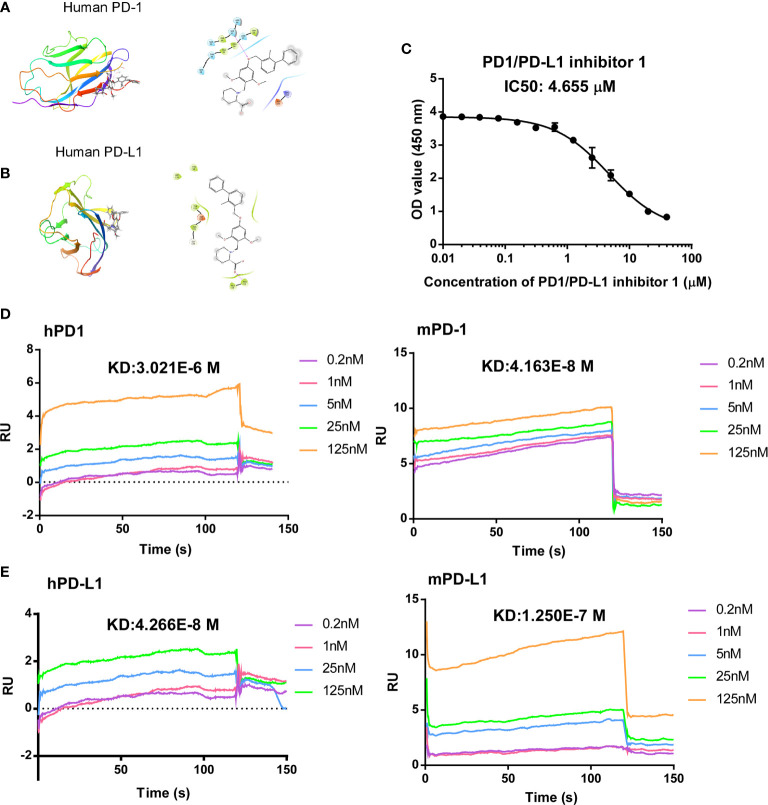
Computer modeling of predicted antagonism of PD-1/PD-L1 binding by PDI-1. **(A, B)** Ribbon representations of a predicted binding pocket for PDI-1 in human PD-1 **(A)** and human PD-L1 **(B)**. Magnified views of the side-chain of Ser567 **(A)** and Phe19 **(B)** with PDI-1 are shown on the right. **(C)** PD-1/PD-L1 binding by PDI-1 *in vitro*, measured using a competitive binding ELISA assay. Data represent the mean ± SEM of three replicates. **(D, E)** Surface plasmon resonance assay of binding of PDI-1 to chip-bound purified human and mouse PD-1 **(D)** or PD-L1 **(E)**.

PDI-1 was first reported by BMS (CN105705489A) and has been identified as a potent and selective small-molecule inhibitor that blocks the interaction between PD-1 and PD-L1. However, the *in-vivo* antagonist effect of PDI-1 to the PD-1/PD-L1 checkpoint still under investigation, so we selected PDI-1, an hPD-L1 inhibitor, as a candidate antagonist. Modeling of PDI-1 docking to hPD-1 and hPD-L1 (**Figures 1A, B**), demonstrates that PDI-1 may interact with the side-chain of serine 57 in hPD-1 and phenylalanine 19 in hPD-L1.

To determine the effectivity of PDI-1 on PD-1/PD-L1 binding, we performed a competitive ELISA assay with solid-phase hPD-1 and solution-phase hPD-L1. We found that PDI-1 dose-dependently inhibited hPD-1 binding to solid-phase PD-L1 with a 50% inhibitory concentration of 4.655 μM (**Figure 1C**). We next investigated whether PDI-1 could bind to human and/or mouse (m) PD-1 and/or PD-L1 using chip-immobilized purified proteins and surface plasmon resonance assays. Interestingly, we found that PDI-1 could bind directly to all four molecules, but had the highest affinity for hPD-L1 and mPD-1 (**Figures 1D, E**). The equilibrium dissociation constants of PDI-1 were 3.021 × 10^−6^ M for hPD-1, 4.163 × 10^−8^ M for mPD-1, 4.266 × 10^−8^ M for hPD-L1, and 1.250 × 10^−7^ M for mPD-L1. Thus, PDI-1 binds to PD-1 and PD-L1 from both species and inhibits the hPD-1/hPD-L1 interaction *in vitro*.

### PDI-1 Increases Anti-Tumor Cytotoxicity and Cytokine Production of T Cells *Ex Vivo*

**Figure 2:**
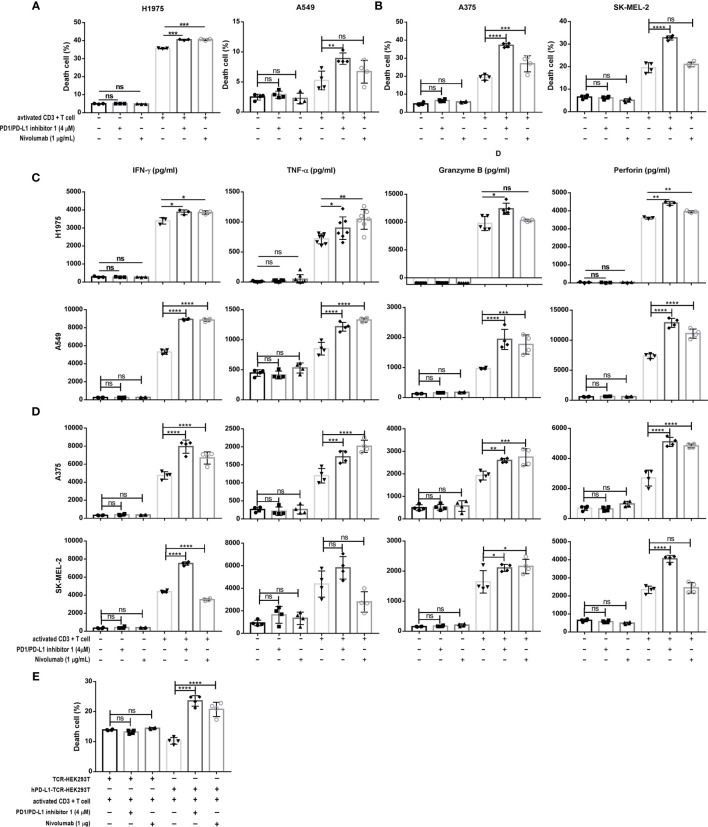
PDI-1 restores T cell cytotoxicity and cytokine production. **(A, B)** Cytotoxicity assay of activated CD3+ T cells cultured for 18 h at an effector to target ratio of 5 to 1 with CFSE-labeled H1975, A549 **(A)** A375, or SK-MEL-2 **(B)** cells alone or in the presence of either PDI-1 (4 μM), nivolumab (1 μg/ml), or an isotype control Ab. At the end of the incubation, cells were labeled with PI and dead or dying tumor cells (CFSE+ PI+) were quantified by flow cytometry. **(C, D)** Cytokine production by activated CD3+ T cells incubated for 18 h, as described for **(A, B)**. Culture supernatants were collected and levels of IFN-γ, TNF-α, perforin, and granzyme B were measured by ELISA assay. **(E)** Activated CD3+ T cells were cultured for 18 h with hPD-L1-TCR-HEK293T cells or TCR-HEK293 T cells in the presence or absence of PDI-1 (4 μM) or nivolumab (1 μg/ml). Dead/dying HEK293 cells were quantified by flow cytometry as described for **(A, B)**. Data represent the mean ± SEM of three replicates. *p < 0.05; **p < 0.01; ***p < 0.001; ****p < 0.0001; ns, not significant.

We then investigated the effects of PDI-1 on the anti-tumor activity of purified human CD3+ T cells *ex vivo*. To confirm that PDI-1 was not directly cytotoxic to human T cells or tumor cells, we examined the effects of incubation with up to 10 μM PDI-1 for 24 hours on the viability of activated human CD3+ T cells (incubated with anti-CD3/CD28-coupled beads for 2 days), two human NSCLC cell lines, NCI-H1975 and A549, and two human melanoma cell lines, A375 and SK-MEL-2. Importantly, we detected no significant effects of PDI-1 on the viability of these cells over the 24- hours incubation (**Supplementary Figures 1A–C**). To measure the effects of PDI-1 on T cell cytotoxicity, activated CD3+ T cells were incubated for 18 h with CFSE-labeled NCI-H1975, A549, A375, and SK-MEL-2 cells at a ratio of 5:1 (E:T) in the presence or absence of PDI-1 or the anti-PD-1 mAb nivolumab. After 18 h co-culture, the cells were labeled with PI and subjected to flow cytometry quantify live (CFSE+) and dead or dying (CFSE+ PI+) cancer cells. We found that T cell cytotoxicity against all four cancer cell lines was significantly higher in the presence of 4 μM PDI-1 compared with vehicle (**Figures 2A, B**). To determine whether PDI-1 also inhibits tumor-cell-induced cytokine production by activated CD3+ T cells, we collected the culture supernatants after 18 h incubation and performed ELISAs to measure secretion of the cytolytic proteins granzyme B and perforin and the inflammatory cytokines IFN-*γ* and TNF-α. As expected, secretion of all four mediators was induced by incubation of T cells with the cancer cells alone, but a striking increase in mediator secretion was detected after treatment with PDI-1 compared with vehicle (**Figures 2C, D**).

To confirm that PDI-1-mediated enhancement of TCR-triggered apoptosis was dependent on disruption of PD-1/PD-L1 interactions, we incubated activated CD3+ T cells with HEK293T cells co-expressing a TCR activator with or without hPD-L1 (hPD-L1-TCR-HEK293T and TCR-HEK293T cells, respectively; **Supplementary Figure 1D**). Co-expression of PD-L1 reduced the ability of activated T cells to induce TCR-HEK293T cell apoptosis, as expected. However, incubation with PDI-1 reversed the PD-L1-mediated suppression of apoptosis, and the level of hPD-L1-TCR-HEK293T cell death exceeded that of TCR-HEK293T cells (**Figure 2E**).

### PDI-1 Promotes T Cell Activation *via* the CD28-TCR Axis

**Figure 3:**
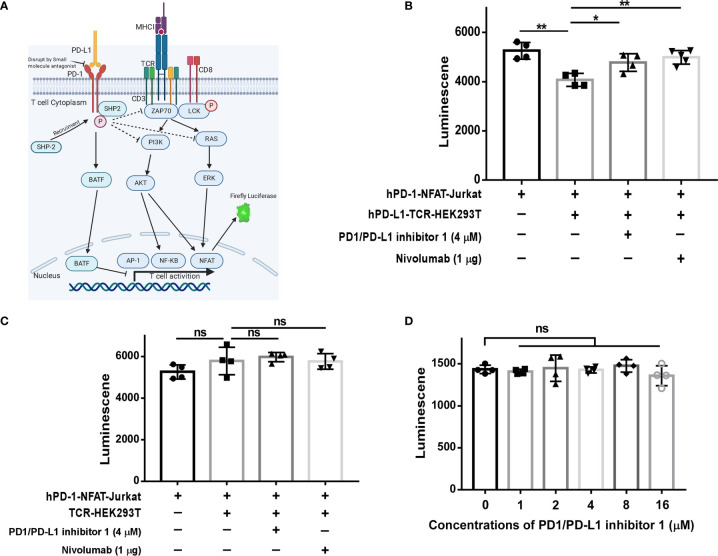
PDI-1 reverses inhibition of NFAT activity by PD-1/PD-L1. **(A)** Schematic of the principle underlying the luciferase reporter assay, in which luciferase expression in Jurkat cells was driven by the NFAT promoter. **(B, C)** hPD-1-NFAT-Jurkat cells were cultured alone or with hPD-L1-TCR-HEK293T cells **(B)** or TCR-HEK293T cells **(C)** overnight. PDI-1 or nivolumab was then added to the cells and luciferase activity was measured after 6 h. **(D)** hPD-1-NFAT-Jurkat cells were cultured with PDI-1 alone for 6 h and luciferase activity was measured. Data represent the mean ± SEM of three replicates. *p < 0.05; **p < 0.01; ns, not significant.

As shown in **Figure 3A**, engagement of the antigen-specific TCR and CD28 costimulatory molecule by tumor cells or antigen-presenting cells triggers a T cell signaling cascade involving ZAP70, PI3K, and RAS pathways (7). Ultimately, this pathway activates the transcription factors NF-κB, AP-1, and NFAT and increases the production of inflammatory cytokines (20). However, activation of this pathway is blocked when the PD-1/PD-L1 axis is engaged (21). Therefore, we next investigated whether PDI-1 enhances T cell activation by blockingPD-1/PD-L1 inhibitory signals. To this end, we employed an assay in which Jurkat T cells were transfected with hPD-1 and an NFAT luciferase reporter (hPD-1-NFAT-Jurkat cells) and incubated with HEK293T cells expressing a TCR activator alone (TCR-HEK293T) or co-expressing hPD-L1 and a TCR activator (hPD-L1-TCR-HEK293T). This system thus serves as a reporter of the effects of PD-1/PD-L1 modulators on TCR signaling. As shown in **Figure 3B**, co-culture of hPD-1-NFAT-Jurkat cells with hPD-L1-TCR-HEK293T cells for 6 h significantly reduced the baseline activity of NFAT. However, addition of either PDI-1 or nivolumab to the culture significantly reversed the inhibition of NFAT activity induced by hPD-L1-TCR-HEK293T cells. This rescue effect of PDI-1 was dependent on PD-1/PD-L1 engagement, because PDI-1 had no effect on NFAT activity in hPD-1-NFAT-Jurkat cells cultured with TCR-HEK293T cells, which lack hPD-L1 expression (**Figure 3C**). Finally, we verified that these results could not simply be explained by a toxic effect by demonstrating that PDI-1 had no effect on the viability of hPD-1-NFAT-Jurkat cells alone (**Figure 3D**). These results indicate that PDI-1 augments T cell activation by preventing PD-1/PD-L1-triggered inhibition of the TCR/CD28-mediated signaling cascade.

### PDI-1 Inhibits the Growth of Melanoma and NSCLC Tumors *In Vivo*

**Figure 4:**
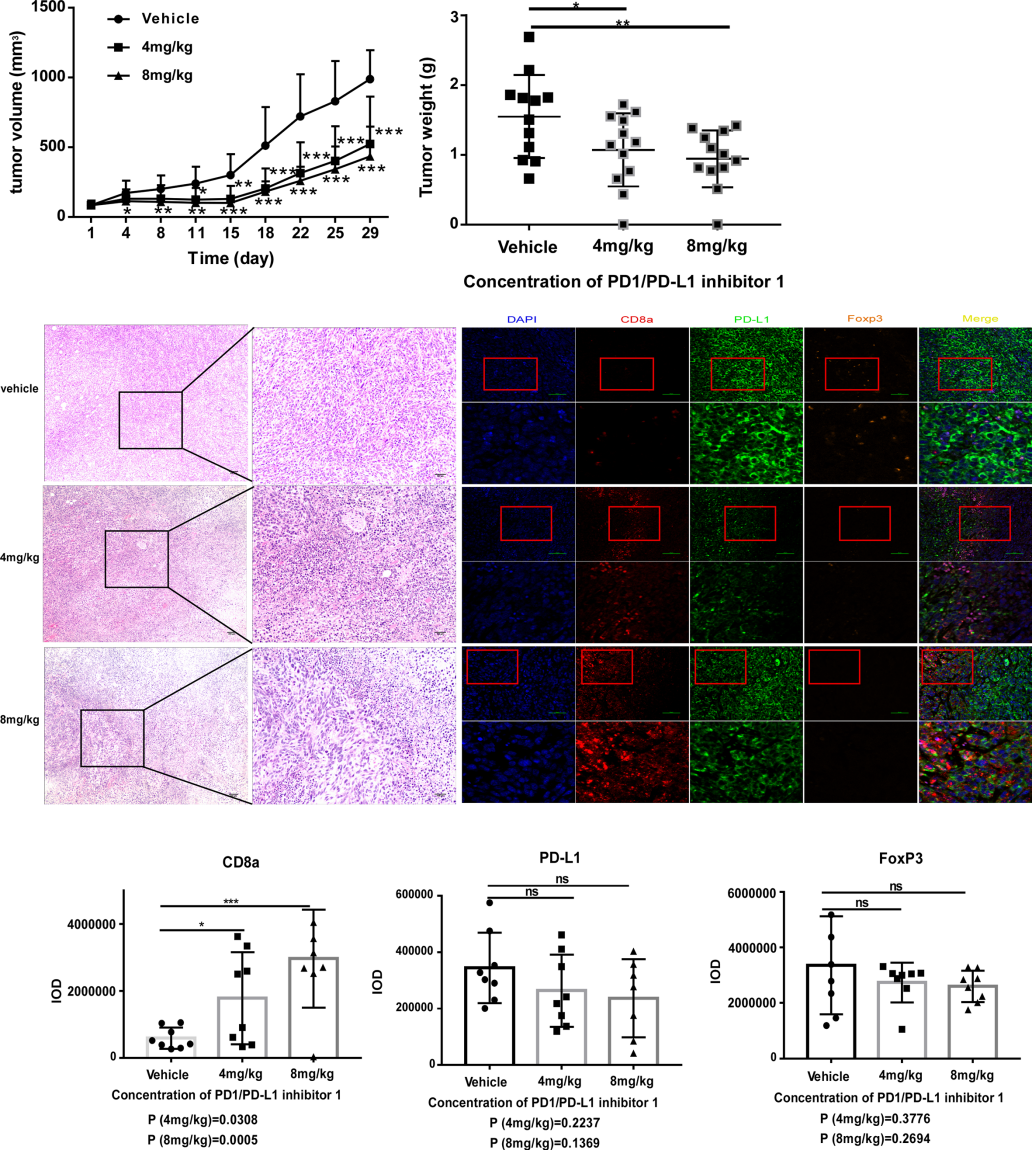
PDI-1 inhibits the growth of human PD-L1-transfected B16-F10 melanoma cells in a mouse model. **(A–D)** B16F10 murine melanoma cells were transfected with hPD-L1 and injected subcutaneously into the flanks of three groups of syngeneic C57BL/6J mice (n=11/group). The mice were then injected intraperitoneally once daily with vehicle, 4 mg/kg PDI-1, or 8 mg/kg PDI-1. **(A, B)** Tumor volumes were measured for up to 29 days. **(B)** Tumors were excised on day 29 and weighed. **(C)** Left: Representative images of H&E-stained tumor sections on day 29. Right: Fluorescent multiplex immunohistochemical staining of PD-L1, CD8a, and FoxP3 proteins in tumors excised on day 29. Nuclei were stained with DAPI. **(D)** Quantification of CD8a, PD-L1, and FoxP3 staining intensity in the images shown in **(C)**. Data represent the mean ± SEM of three replicates. *p < 0.05; **p < 0.01; ***p < 0.001; ns, not significant.

**Figure 5:**
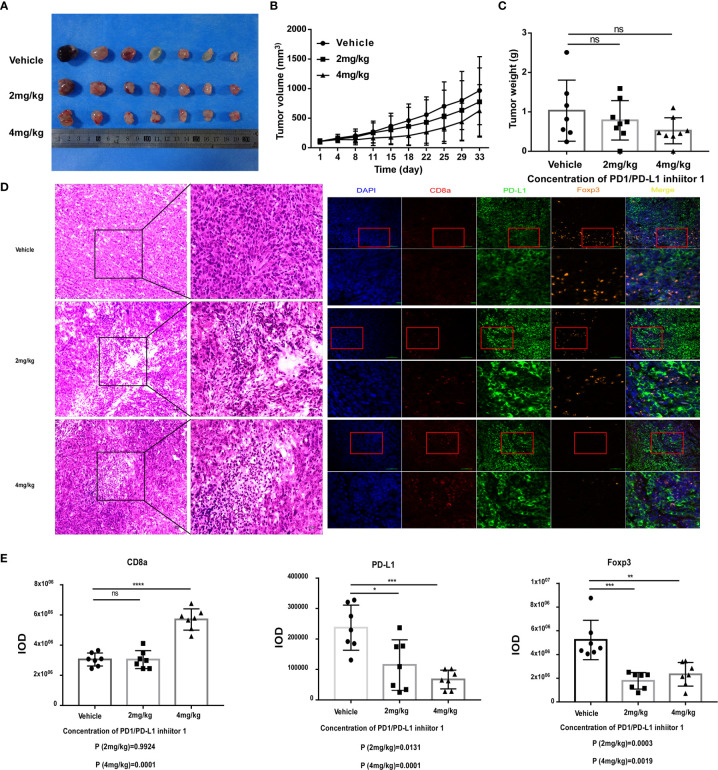
PDI-1 inhibits the growth of human PD-L1-transfected KLN205 NSCLC cells in a mouse model. **(A–E)** KLN205 cells were transfected with hPD-L1 and injected subcutaneously into the flanks of three groups of syngeneic DBA/2 mice (n=8/group). The mice were then injected intraperitoneally once daily with vehicle, 2 mg/kg PDI-1, or 4 mg/kg PDI-1. **(A)** Representative photographs of tumors excised on day 33. **(B)** Tumor volumes measured for up to 33 days. **(C)** Tumor weights on day 33. **(D)** Left: Representative images of H&E-stained tumor sections on day 33. Right: Fluorescent multiplex immunohistochemical staining of PD-L1, CD8a, and FoxP3 proteins in tumors excised on day 33. Nuclei were stained with DAPI. **(E)** Quantification of CD8a, PD-L1, and FoxP3 staining intensity in the images shown in **(D)**. Data represent the mean ± SEM of three replicates. *p < 0.05; **p < 0.01; ***p < 0.001; ns, not significant.

Having demonstrated that PDI-1 effectively blocks PD-1/PD-L1-mediated suppression of anti-tumor cytotoxicity and cytokine production by human T cells *ex vivo*, we next determined whether PDI-1 would have beneficial effects on tumor growth *in vivo*. We transfected the mouse NSCLC cell line KLN205 and the mouse melanoma cell line B16F10 with hPD-L1 and verified that the transfectants expressed much higher levels of hPD-L1 relative to the levels of endogenous mPD-L1 (**Supplementary Figure 2**). To determine whether PDI-1 was directly toxic to mice, groups of wild-type C57BL/6 mice were injected intraperitoneally with vehicle or 2, 4, or 8 mg/kg PDI-1 once daily for 13-days. As shown in **Supplementary Figure 3**, there were no detectable effects of PDI-1 treatment on the liver, spleen, or body weights of the treated mice. To examine the effects of PDI-1 treatment on tumor growth, groups of C57BL/6 mice (B16-F10 syngeneic) were subcutaneously injected with hPD-L1-B16-F10 melanoma cells (**Figure 4**) or groups of DBA/2 mice (KLN205 syngeneic) were injected subcutaneously with hPD-L1-KLN205 cells (**Figure 5**), and the mice were injected intraperitoneally with the vehicle, 4 mg/kg PDI-1, or 8 mg/kg PDI-1 once daily for either 29 days (C57BL/6) or 33 days (DBA/2). Compared with vehicle-treated mice, PDI-1 treatment dramatically attenuated the growth of hPD-L1-B16F10 tumors (**Figures 4A, B**, **Supplementary Figure 4A**), and a similar trend, albeit not statistically significant, was observed for the effect of PDI-1 on the growth of hPD-L1-KLN205 tumors (**Figures 5A–C**). Importantly, the spleen, liver, and body weights of the tumor-bearing mice were not affected by PDI-1 treatment compared with vehicle treatment (**Supplementary Figures 4B, C**). At the end of the experiment (days 29 or 33), the tumors were excised for further analysis. H&E staining of hPD-L1-B16-F10 tumors (**Figure 4C**) and hPD-L1-KLN205 tumors (**Figure 5D**), showed that PDI-1 treatment caused a marked increase in tumor infiltration by inflammatory cells and necrosis of tumor cells compared with tumors from vehicle-treated mice. The abundance of CD8+ T cells and FoxP3+ T regulatory cells (Tregs), as well as PD-L1 expression by tumor and stromal cells in the tumor microenvironment (TME), are predictors of prognosis and response to immunotherapy in human NSCLC and melanoma (22). Fluorescent multiplex immunohistochemical (mIHC-F) staining of CD8a, FoxP3, and PD-L1 proteins in sections of hPD-L1-B16-F10 tumors (**Figures 4C, D**) demonstrated that PDI-1 treatment resulted in an increase in CD8+ T cells, decrease in FoxP3+ Tregs, and reduction in PD-L1 expression compared with vehicle treatment, although only the effects of PDI-1 on CD8+ T cells abundance reached the level of statistical significance (**Figure 4D**). In contrast, mIHC-F of hPD-L1-KLN205 tumor sections showed highly significant increases in CD8+ T cells and reductions in FoxP3+ Tregs and PD-L1 expression in PDI-1-treated mice compared with vehicle-treated mice (**Figures 5D, E**). These results demonstrate that PDI-1 treatment inhibits the growth of NSCLC and melanoma tumor cells, likely through a mechanism involving increased recruitment/activation of cytotoxic T cells and reduced recruitment/activation of inhibitory Tregs.

### PDI-1 Rapidly Promotes Activation of the Anti-Tumor T Cell Response *In Vivo*

**Figure 6:**
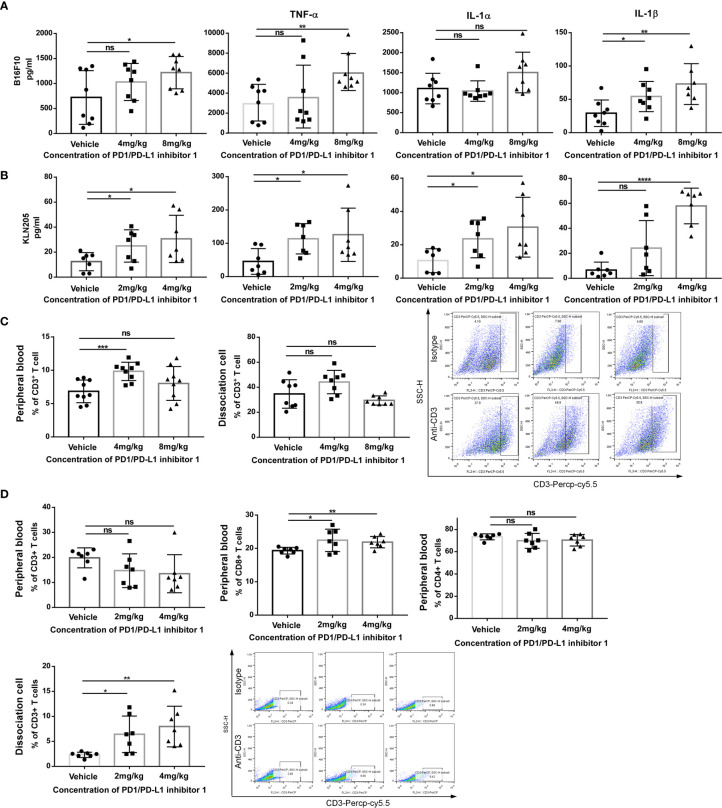
PDI-1 increases T cell-mediated anti-tumor responses *in vivo.* **(A, B)** Blood samples were collected on day 29 from hPD-L1-B16F10 tumor-bearing mice **(A)** or on day 33 from hPD-L1-KLN205 tumor-bearing mice, as described in **Figures 4** and **5**. Serum levels of IFN-γ, TNF-α, IL-1α, and IL-1β were analyzed using a multiplex flow cytometry assay. **(C)** Flow cytometric analysis of CD3+ T cells in peripheral blood mononuclear cells (left) or dissociated tumor tissue (middle) on day 29 after injection of hPD-L1-B16F10 cells. Dot plots (right) show representative FACS analysis of CD3 + T cells derived from tumor specimens. **(D)** Flow cytometric analysis of CD3+, CD4+, and CD8+ T cells in peripheral blood or of intratumorally CD3+ T cells on day 33 after injection of hPD-L1-KLN205 cells. Dot plots show representative FACS analysis of CD3 + T cells derived from tumor specimens. Data represent the mean ± SEM of three replicates. *p < 0.05; **p < 0.01; ***p < 0.001; ****p < 0.0001; ns, not significant.

To investigate the immune mechanism of action of PDI-1 in more detail, we compared the serum cytokine content of tumor-bearing mice on day 29 (hPD-L1-B16-F10) or day 33 (hPD-L1-KLN205) after treatment with vehicle or PDI-1. Using a multiplex flow cytometry assay, we measured serum concentrations of IL-1α, IL-1β, IL-6, IL-10, IL-12p70, IL-17A, IL-23, IL-27, monocyte chemotactic protein-1 (MCP-1), interferon (IFN)-β, IFN-γ, tumor necrosis factor-α (TNF-α), and granulocyte macrophage-colony stimulating factor (GM-CSF). We found that PDI-1 significantly increased serum levels of several cytokines, most notably IFN-γ, IFN-β, IL-1α, and IL-1β, compared with vehicle (**Figures 6A, B**). We also analyzed the abundance of total CD3+ T cells and/or CD4+ and CD8+ T cell subsets in the peripheral blood and dissociated tumor tissues by flow cytometry. In hPD-L1-B16-F10 tumor-bearing mice, treatment with 4 mg/kg PDI-1 significantly increased the percentage of CD3+ T cells in the peripheral blood compared with vehicle-treated mice, and there was a similar, albeit not significant, trend in the effects of PDI-1 on the abundance of intratumoral CD3+ T cells (**Figure 6C**). Interestingly, treatment with PDI-1 at 8 mg/kg had a smaller effect than 4 mg/kg PDI-1 on CD3+ T cell percentages in both the peripheral blood and tumor tissue (**Figure 6C**). In the PD-L1-KLN205 tumor-bearing mice, the effects of PDI-1 on peripheral blood T cells were more variable than in hPD-L1-B16-F10-bearing mice, although the percentage of CD8+ cells was significantly higher in PDI-1-treated mice than in vehicle-treated mice (**Figure 6C**). Notably, the percentage of intratumoral CD3+ T cells was significantly and dose-dependently increased in hPD-L1-KLN205 tumors from PDI-1-treated compared with vehicle-treated mice (**Figure 6D**).

### PDI-1 Rapidly Boosts the Host Anti-Tumor Immune Response

**Figure 7:**
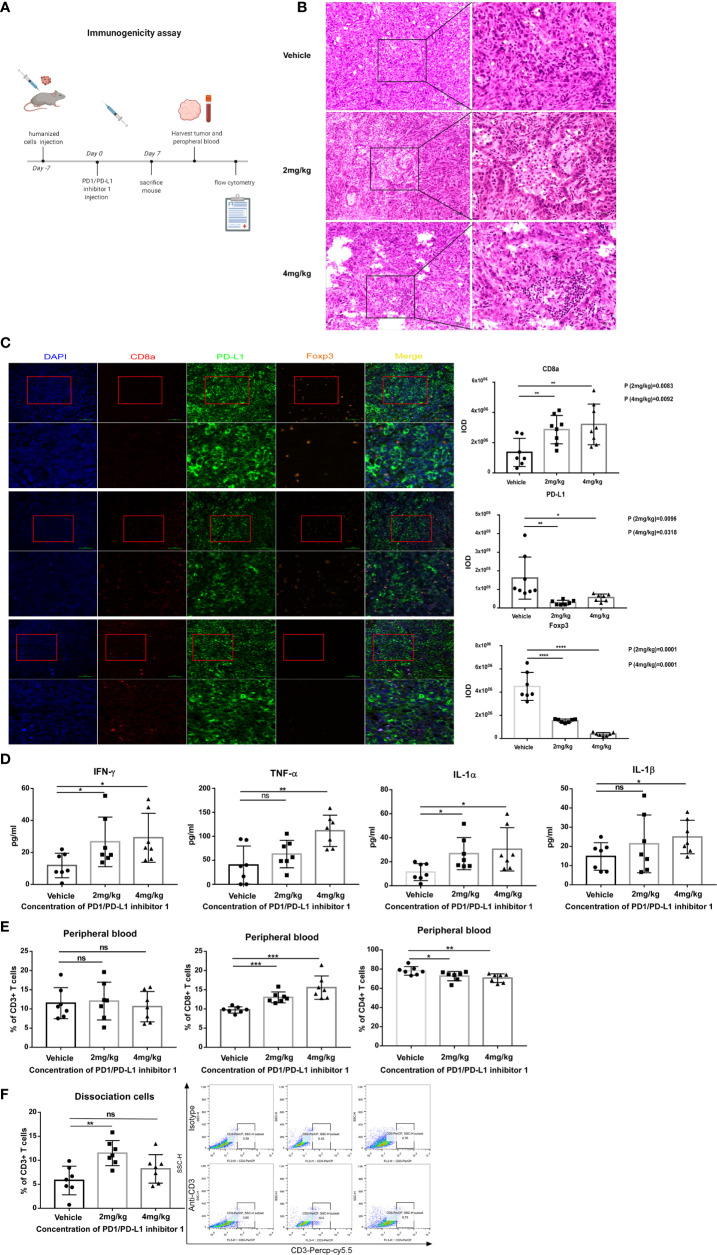
PDI-1 treatment rapidly boosts the host immune response to hPD-L1-KLN205 NSCLC tumors. **(A)** Schematic of the experimental design. DBA/2 mice were injected subcutaneously with hPD-L1-KLN205 cells and administered vehicle, 2 mg/kg PDI-1, or 4 mg/kg PDI-1 by intraperitoneal injection. Mice were sacrificed on day 7 and peripheral blood and tumors were collected for analysis. **(B)** Representative images of H&E-stained tumor sections. **(C)** Fluorescent multiplex immunohistochemical staining of CD8a, PD-L1, and FoxP3 protein in excised tumors. Nuclei were stained with DAPI. **(D)** Serum levels of IFN-γ, TNF-α, IL-1α, and IL-1β were analyzed using a multiplex flow cytometry assay. **(E, F)** Flow cytometric analysis of CD3+, CD4+, and CD8+ T cells in peripheral blood **(E)** or of intratumoral CD3+ T cells **(F)** on day 7. Dot plots indicate representative FACS analysis of CD3 + T cells derived from tumor specimens. Data represent the mean ± SEM of three replicates. *p < 0.05; **p < 0.01; ***p < 0.001; ns, not significant.

Because the analyses of tumor-infiltrating T cells in the preceding section were performed on days 29 and 33, we next asked whether the effects of PD-L1 blockade might be apparent much earlier in the response. The experiments were therefore repeated with hPD-L1-KLN205 tumors, and the mice were sacrificed on day 7 (**Figure 7A**). Indeed, tumors excised from PDI-1-treated mice displayed enhanced necrosis of cancer cells and infiltration by inflammatory cells compared with tumors from vehicle-treated mice (**Figure 7B**). Furthermore, mIHC-F staining showed that PDI-1 caused a marked increase in CD8a expression and a decrease in PD-L1 and FoxP3 expression in hPD-L1-KLN205 tumors (**Figure 7C**). Evaluation of serum cytokine levels revealed elevated levels of inflammatory cytokines, including IL-1α, IL-1β, IFN-γ, and TNF-α, in PDI-1-treated compared with vehicle-treated mice (**Figure 7D**). Finally, we analyzed the percentage of CD3+, CD4+, and/or CD8+ T cells in the peripheral blood and dissociated tumor tissues of the treatment mice (**Figures 7E, F**). Although PDI-1 treatment did not affect the percentage of total CD3+ T cells in peripheral blood, the relative abundance of CD8+ and CD4+ T cells was increased and decreased, respectively, in PDI-1-treated mice compared with vehicle-treated mice (**Figure 7E**). In addition, treatment with 4 mg/kg PDI-1 caused a significant increase in the abundance of tumor-associated CD3+ T cells (**Figure 7F**). Importantly, the beneficial anti-cancer effects of PDI-1 on T cell abundance, cytokine production, and PD-L1 expression were as good as or superior to the effects of the anti-PD-L1 mAb atezolizumab (**Supplementary Figures 7A–C**).

## Discussion

Accumulating preclinical and clinical data support the crucial role of immune checkpoint pathways as therapeutic targets for cancer. Elucidation of the molecular mechanisms of PD-1 activity and the identification of its ligands (23, 24) has enabled detailed investigation of the involvement of this receptor–ligand pair in the anti-tumor immune response. As noted earlier, PD-1/PD-L1 interactions play a physiological role in maintaining tolerance through suppression of T cell activation, and expression of PD-L1 by cancer cells enables them to evade host immune surveillance (25). Several anti-PD-1 mAbs have been developed and approved for cancer therapy, including nivolumab, pembrolizumab, and atezolizumab (26); nevertheless, about 70% of patients with small cell lung cancer do not respond well to anti-PD-1 mAbs (27). The reason for such unresponsiveness is unclear and is under intense investigation.

Although small molecule blockers of the PD-1/PD-L1 interaction would have intrinsic advantages over mAbs, their development has progressed more slowly. In addition to the benefits already described, small molecules have the potential to enter cells and thus target the molecular mechanisms of immunosuppression not accessible to mAb therapies (28). Thus, novel small molecule compounds that could be used alone or in combination with mAbs provide an alternative therapeutic approach for patients with poor clinical responses and/or resistance to existing drugs.

In this study, we identified PDI-1 as a small molecule that binds to hPD-1 and hPD-L1 and disrupts their interactions *in vitro* and *in vivo*. Nivolumab binds to hPD-1 at amino acids 25–34 (29), whereas our docking simulations suggest that PDI-1 binds in the region of serine 57 of hPD-1. Thus, the binding sites of nivolumab and PDI-1 are non-overlapping and the combination of both agents could potentially provide superior efficacy compared with either agent alone.

We demonstrated that PDI-1 blocks PD-1/PD-L1-mediated negative regulation of TCR-activated T cells (30), thereby promoting T cell cytotoxicity and cytokine production, cancer cell apoptosis, and recruitment of inflammatory cells to the TME (31, 32). In syngeneic mouse tumor models, PDI-1 inhibited the growth of hPD-L1-expressing melanoma and NSCLC by increasing the abundance of effector CD8+ T cells and decreasing that of inhibitory FoxP3 CD4+ Tregs. We also verified that the PD-1/PD-L1 axis contributes to tumor immune evasion, and our results suggest that PDI-1 may rejuvenate immunosurveillance by relieving T cell anergy induced by potent and chronic stimulation by tumor antigens (33). PD-1 engagement by PD-L1 on cancer cells is known to interfere with TCR/CD28-dependent T cell signaling by blocking exhaustion-associated transcription factors, including the AP-1, NF-κB, and NFAT (34). Martinez et al. showed that different *styles of NFAT* trigger transcriptional profiles associated with exhausted *versus* effector T cell phenotypes (35). PD-L1 binding to PD-1 induces phosphorylation of the immunoreceptor tyrosine switch motif in the cytoplasmic domain of PD-1, which leads to recruitment of SHP2 phosphatase, inhibition of downstream signaling through ZAP70 and PI3K, and inhibition of T cell proliferation, differentiation, and cytokine production (36–38). Consistent with this, we demonstrated that PDI-1 enhances NFAT activity in Jurkat T cells in a TCR- and PD-L1-dependent manner, thus providing a molecular mechanism by which PDI-1 enhances anti-tumor responses to NSCLC and melanoma in mice. Importantly, treatment of mice with PDI-1 alone did not affect organ or body weights, suggesting that PDI-1 has a favorable safety profile. We detected the distribution of PDI-1 in mice (**Supplementary Figure 8**). The MRM chromatograms in **Supplementary Figure 8** show the serum concentrations of PDI-1 increase at 5 minutes.

Our finding, that PDI-1 is efficacious and safe in two *in vivo* models of cancer, suggests that this compound could be clinically developed as a new ICI. Small molecule compounds represent an important potential source of novel anti-cancer drugs; however, few small molecules affecting immune regulation have been developed to date. Further studies on the immunomodulatory activity of compounds such as PDI-1 will be of considerable importance in advancing the development of small molecule anti-cancer drugs.

## Data Availability Statement

The original contributions presented in the study are included in the article/**Supplementary Material**, further inquiries can be directed to the corresponding author/s.

## Ethics Statement

The animal study was reviewed and approved by the Ethics Committee of China-US (Henan) Hormel Cancer Institute (CUHCI2019001).

## Author Contributions

YW, TG and M-HL were involved in study concept and design, acquisition of data, analysis, and interpretation of data, and drafting of the manuscript. YW, TG, XT, WL, RZ and WY performed the experiments. QG, TL, CZ, J-HS, and KL provided material support. YW, TG and M-HL wrote the manuscript. M-HL supervised the study. All authors contributed to the article and approved the submitted version.

## Funding

This work was supported by grant funding from the National Natural Science Foundation of China NSFC81972839, and the Key Program of Henan Province, China Grant NO.161100510300 and Henan Provincial Government, China.

## Conflict of Interest

The authors declare that the research was conducted in the absence of any commercial or financial relationships that could be construed as a potential conflict of interest.

## Supplementary Material

Supplementary Figure 1 PDI-1 is not toxic to cancer cells or CD3+ T cells. **(A–C)** Proliferation of NCI-H1975, A549 **(A)** A375, SK-MEL-2 **(B)** tumor cells or CD3+ T cells **(C)**. Cells were cultured overnight and then incubated with the indicated concentrations of PDI-1 for an additional 24 hours. Viability was measured using an MTS assay. **(D)** Flow cytometric analysis of hPD-L1 expression on TCR-HEK293T and hPD-L1-TCR-HEK293T cells. Data represent the mean ± SEM of three replicates. **p* < 0.05; ***p* < 0.01; ****p* < 0.001.

Supplementary Figure 2 Mouse and human PD-L1 expression in transfected cancer cell lines. (A, B) Flow cytometric analysis of double staining of human (h) and mouse (m) PD-L1 on murine KLN205 NSCLC cells **(A)** and murine B16-F10 melanoma cells **(B)** after transfection with human PD-L1. Cells were stained with hPD-L1 or an isotype control IgG antibody.

Supplementary Figure 3 PDI-1 exhibits no toxicity in wild-type C57BL/6 mice. **(A–C)** Groups of 5–6-week-old wild-type C57BL/6 mice were administered vehicle or the indicated doses of PDI-1 by intraperitoneal injection once daily for 13 days. Liver **(A)**, spleen **(B)** and body **(C)** weights were determined on day 13. Data represent the mean ± SEM of 6 mice/group. ns, not significant.

Supplementary Figure 4 PDI-1 exhibits no toxicity in mice bearing hPD-L1-transfected melanoma or NSCLC tumors. **(A, B)** Groups of C57BL/6 mice (n=11) were injected with hPD-L1-B16-F10 melanoma cells and administered vehicle or the indicated doses of PDI-1 by intraperitoneal injection once daily for 29 days. Tumors were excised and photographed **(A)** and the liver, spleen, and body weights **(B)** were then determined. **(C)** Groups of DBA/2 mice (n=8) were injected with hPD-L1-KLN205 NSCLC cells and administered vehicle or the indicated doses of PDI-1 by intraperitoneal injection once daily for 33 days. Liver, spleen, and body weights were then determined. Data represent the mean ± SEM. ns, not significant.

Supplementary Figure 5 PDI-1 increases inflammatory cytokine production in hPD-L1-transfected tumor-bearing mice. **(A, B)** Blood samples were collected from C57BL/6 mice bearing hPD-L1-B6F10 tumors on day 29 **(A)** or from DBA/2 mice bearing hPD-L1-KLN205 tumors on day 33 **(B)**. Sera were analyzed for the indicated inflammatory mediators using a multiplex flow cytometry assay. Data represent the mean ± SEM of 8 mice/group. **p *< 0.05; ***p *< 0.01; ****p *< 0.001; *****p *< 0.0001; ns, not significant.

Supplementary Figure 6 PDI-1 rapidly boosts the host immune response to hPD-L1-bearing tumor cells. DBA/2 mice harboring hPD-L1-KLN205 tumors were bled on day 7 and sera were analyzed for the indicated inflammatory mediators using a multiplex flow cytometry assay. Data represent the mean ± SEM of 7 mice/group. **p *< 0.05; ***p *< 0.01; ns, not significant.

Supplementary Figure 7 Comparison of PDI-1 with Atezolizumab in an animal model. **(A–C)** Groups of C57BL/6 (n=6) mice were injected with hPD-L1-B16-F10 murine melanoma cells and administered vehicle, 4 or 8 mg/kg PDI-1, or the anti-PD-L1 mAb atezolizumab (10 mg/kg) by intraperitoneal injection once daily for 7 days. Tumors and blood samples were collected on day 14. **(A)** Representative images of H&E-stained tumor sections. **(B)** Fluorescent multiplex immunohistochemical staining of PD-L1, CD8a, and FoxP3 protein in excised tumors. **(C)** Serum levels of the indicated cytokines were determined using a multiplex flow cytometry assay. **p* < 0.05; ***p* < 0.01; ****p* < 0.001; *****p* < 0.0001; ns, not significant.

Supplementary Figure 8 Distribution of PDI-1 in mice. MRM chromatograms for PDI-1 detected in the serum of C57BL/C male mice.

## References

[1] Topalian SL Weiner GJ Pardoll DM. Cancer Immunotherapy Comes of Age. J Clin Oncol: Off J Am Soc Clin Oncol (2011) 29(36):4828–36. 10.1200/JCO.2011.38.0899 PMC3255990 22042955

[2] Hanahan D Weinberg RA. Hallmarks of Cancer: The Next Generation. Cell (2011) 144(5):646–74. 10.1016/j.cell.2011.02.013 21376230

[3] Seliger B. Basis of PD1/PD-L1 Therapies. J Clin Med (2019) 8(12):2168. 10.3390/jcm8122168 PMC6947170 31817953

[4] Mahoney KM Rennert PD Freeman GJ. Combination Cancer Immunotherapy and New Immunomodulatory Targets. Nat Rev Drug Discovery (2015) 14(8):561–84. 10.1038/nrd4591 26228759

[5] Lesokhin AM Callahan MK Postow MA Wolchok JD. On Being Less Tolerant: Enhanced Cancer Immunosurveillance Enabled by Targeting Checkpoints and Agonists of T Cell Activation. Sci Trans Med (2015) 7(280):280sr1. 10.1126/scitranslmed.3010274 25810313

[6] Wang X Teng F Kong L Yu J. Pd-L1 Expression in Human Cancers and its Association With Clinical Outcomes. OncoTargets Ther (2016) 9:5023–39. 10.2147/OTT.S105862 PMC4990391 27574444

[7] Ostrand-Rosenberg S Horn LA Haile ST. The Programmed Death-1 Immune-Suppressive Pathway: Barrier to Antitumor Immunity. J Immunol (Baltimore Md 1950) (2014) 193(8):3835–41. 10.4049/jimmunol.1401572 PMC4185425 25281753

[8] Ai L Xu A Xu J. Roles of PD-1/PD-L1 Pathway: Signaling, Cancer, and Beyond. Adv Exp Med Biol (2020) 1248:33–59. 10.1007/978-981-15-3266-5_3 32185706

[9] Blackburn SD Shin H Freeman GJ Wherry EJ. Selective Expansion of a Subset of Exhausted CD8 T Cells by PD-L1 Blockade. Proc Natl Acad Sci (2008) 105(39):15016–21. 10.1073/pnas.0801497105 PMC2567485 18809920

[10] Pauken KE Wherry EJ. Overcoming T Cell Exhaustion in Infection and Cancer. Trends Immunol (2015) 36(4):265–76. 10.1016/j.it.2015.02.008 PMC4393798 25797516

[11] Zhan MM Hu XQ Liu XX Ruan BF Xu J Liao C. From Monoclonal Antibodies to Small Molecules: The Development of Inhibitors Targeting the PD-1/PD-L1 Pathway. Drug Discovery Today (2016) 21(6):1027–36. 10.1016/j.drudis.2016.04.011 27094104

[12] Gomes F Serra-Bellver P Lorigan P. The Role of Nivolumab in Melanoma. Future Oncol (London England) (2018) 14(13):1241–52. 10.2217/fon-2017-0484 29328782

[13] Gettinger S Rizvi NA Chow LQ Borghaei H Brahmer J Ready N. Nivolumab Monotherapy for First-Line Treatment of Advanced non-Small-Cell Lung Cancer. J Clin Oncol: Off J Am Soc Clin Oncol (2016) 34(25):2980–7. 10.1200/JCO.2016.66.9929 PMC5569692 27354485

[14] Reddy SM Carroll E Nanda R. Atezolizumab for the Treatment of Breast Cancer. Expert Rev Anticancer Ther (2020) 20(3):151–8. 10.1080/14737140.2020.1732211 32067545

[15] Santini FC Rudin CM. Atezolizumab for the Treatment of non-Small Cell Lung Cancer. Expert Rev Clin Pharmacol (2017) 10(9):935–45. 10.1080/17512433.2017.1356717 PMC6089509 28714780

[16] Farkona S Diamandis EP Blasutig IM. Cancer Immunotherapy: The Beginning of the End of Cancer? BMC Med (2016) 14(1):73. 10.1186/s12916-016-0623-5 27151159 PMC4858828

[17] Adams JL Smothers J Srinivasan R Hoos A. Big Opportunities for Small Molecules in Immuno-Oncology. Nat Rev Drug Discovery (2015) 14(9):603–22. 10.1038/nrd4596 26228631

[18] Zhu HF Li Y. Small-Molecule Targets in Tumor Immunotherapy. Natural Products Bioprospect (2018) 8(4):297–301. 10.1007/s13659-018-0177-7 PMC6102179 29974338

[19] Vladimer GI Snijder B Krall N Bigenzahn JW Huber KVM Lardeau CH. Global Survey of the Immunomodulatory Potential of Common Drugs. Nat Chem Biol (2017) 13(6):681–90. 10.1038/nchembio.2360 PMC5438060 28437395

[20] Okazaki T Chikuma S Iwai Y Fagarasan S Honjo T. A Rheostat for Immune Responses: The Unique Properties of PD-1 and Their Advantages for Clinical Application. Nat Immunol (2013) 14(12):1212–8. 10.1038/ni.2762 24240160

[21] Arasanz H Gato-Cañas M Zuazo M Ibañez-Vea M Breckpot K Kochan G. PD1 Signal Transduction Pathways in T Cells. Oncotarget (2017) 8:10. 10.18632/oncotarget.17232 PMC5584302 28881701

[22] Morihiro T, Kuroda S, Kanaya N, Kakiuchi Y, Kubota T, Aoyama K T. Pd-L1 Expression Combined With Microsatellite Instability/CD8+ Tumor Infiltrating Lymphocytes as a Useful Prognostic Biomarker in Gastric Cancer. Sci Rep (2019) 9(1):4633. 10.1038/s41598-019-41177-2 30874607 PMC6420501

[23] Freeman GJ Long AJ Iwai Y Bourque K Chernova T Nishimura H. Engagement of the PD-1 Immunoinhibitory Receptor by a Novel B7 Family Member Leads to Negative Regulation of Lymphocyte Activation. J Exp Med (2000) 192(7):1027–34. 10.1084/jem.192.7.1027 PMC2193311 11015443

[24] Latchman Y Wood CR Chernova T Chaudhary D Borde M Chernova I. Pd-L2 is a Second Ligand for PD-1 and Inhibits T Cell Activation. Nat Immunol (2001) 2(3):261–8. 10.1038/85330 11224527

[25] Sun Z Fourcade J Pagliano O Chauvin JM Sander C Kirkwood JM. IL10 and PD-1 Cooperate to Limit the Activity of Tumor-Specific Cd8+ T Cells. Cancer Res (2015) 75(8):1635–44. 10.1158/0008-5472.can-14-3016 PMC4401638 25720800

[26] Freeman-Keller M Kim Y Cronin H Richards A Gibney G Weber JS. Nivolumab in Resected and Unresectable Metastatic Melanoma: Characteristics of Immune-Related Adverse Events and Association With Outcomes. Clin Cancer Res an Off J Am Assoc Cancer Res (2016) 22(4):886–94. 10.1158/1078-0432.ccr-15-1136 PMC4755809 26446948

[27] Topalian SL Hodi FS Brahmer JR Gettinger SN Smith DC McDermott DF. Safety, Activity, and Immune Correlates of Anti-PD-1 Antibody in Cancer. N Engl J Med (2012) 366(26):2443–54. 10.1056/NEJMoa1200690 PMC3544539 22658127

[28] Chen S Song Z Zhang A. Small-Molecule Immuno-Oncology Therapy: Advances, Challenges and New Directions. Curr Topics Medicinal Chem (2019) 19(3):180–5. 10.2174/1568026619666190308131805 30854972

[29] Tan S Zhang H Chai Y Song H Tong Z Wang Q. An Unexpected N-terminal Loop in PD-1 Dominates Binding by Nivolumab. Nat Commun (2017) 8:14369. 10.1038/ncomms14369 28165004 PMC5303876

[30] Butte MJ Keir ME Phamduy TB Sharpe AH Freeman GJ. Programmed Death-1 Ligand 1 Interacts Specifically With the B7-1 Costimulatory Molecule to Inhibit T Cell Responses. Immunity (2007) 27(1):111–22. 10.1016/j.immuni.2007.05.016 PMC2707944 17629517

[31] Scheurich P Thoma B Ucer U Pfizenmaier K. Immunoregulatory Activity of Recombinant Human Tumor Necrosis Factor (TNF)-Alpha: Induction of TNF Receptors on Human T Cells and TNF-alpha-mediated Enhancement of T Cell Responses. J Immunol (Baltimore Md 1950) (1987) 138(6):1786–90. 3029221

[32] Perez C Albert I DeFay K Zachariades N Gooding L Kriegler M. A Nonsecretable Cell Surface Mutant of Tumor Necrosis Factor (TNF) Kills by Cell-to-Cell Contact. Cell (1990) 63(2):251–8. 10.1016/0092-8674(90)90158-b 2208285

[33] Sotomayor EM Borello I Levitsky HI. Tolerance and Cancer: A Critical Issue in Tumor Immunology. Crit Rev Oncogene (1996) 7:433–56. 10.1615/critrevoncog.v7.i5-6.30 9467666

[34] Kahan SM Wherry EJ Zajac AJ. T Cell Exhaustion During Persistent Viral Infections. Virology (2015) 479-480:180–93. 10.1016/j.virol.2014.12.033 PMC4424083 25620767

[35] Martinez GJ Pereira RM Äijö T Kim EY Marangoni F Pipkin ME. The Transcription Factor NFAT Promotes Exhaustion of Activated Cd8+ T Cells. Immunity (2015) 42(2):265–78. 10.1016/j.immuni.2015.01.006 PMC4346317 25680272

[36] Chemnitz JM Parry RV Nichols KE June CH Riley JL. SHP-1 and SHP-2 Associate With Immunoreceptor Tyrosine-Based Switch Motif of Programmed Death 1 Upon Primary Human T Cell Stimulation, But Only Receptor Ligation Prevents T Cell Activation. J Immunol (Baltimore Md: 1950) (2004) 173(2):945–54. 10.4049/jimmunol.173.2.945 15240681

[37] Riley JL. PD-1 Signaling in Primary T Cells. Immunol Rev (2009) 229(1):114–25. 10.1111/j.1600-065X.2009.00767.x PMC3424066 19426218

[38] Sheppard KA Fits LJ Lee JM Benander C George JA Wooters J. PD-1 Inhibits T-cell Receptor Induced Phosphorylation of the ZAP70/CD3zeta Signalosome and Downstream Signaling to Pkctheta. FEBS Lett (2004) 574:37–41. 10.1016/j.febslet.2004.07.083 15358536

