# Supplementary figures and images for: A Small Molecule Antagonist of PD-1/PD-L1 Interactions Acts as an Immune Checkpoint Inhibitor for NSCLC and Melanoma Immunotherapy
Source: Front Immunol. 2021 May 14;12:654463. doi: 10.3389/fimmu.2021.654463 (PMC8160380; doi:10.3389/fimmu.2021.654463)

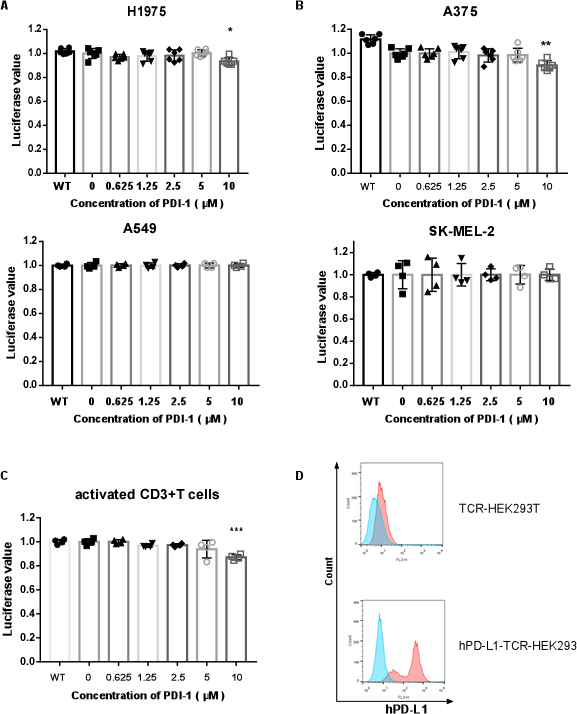

Supplement: Supplementary Figure 1 — PDI-1 is not toxic to cancer cells or CD3+ T cells. (A–C) Proliferation of NCI-H1975, A549 (A) A375, SK-MEL-2 (B) tumor cells or CD3+ T cells (C). Cells were cultured overnight and then incubated with the indicated concentrations of PDI-1 for an additional 24 hours. Viability was measured using an MTS assay. (D) Flow cytometric analysis of hPD-L1 expression on TCR-HEK293T and hPD-L1-TCR-HEK293T cells. Data represent the mean ± SEM of three replicates. *p < 0.05; **p < 0.01; ***p < 0.001. [file Image_1.tif]

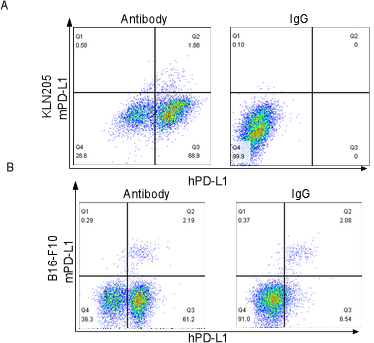

Supplement: Supplementary Figure 2 — Mouse and human PD-L1 expression in transfected cancer cell lines. (A, B) Flow cytometric analysis of double staining of human (h) and mouse (m) PD-L1 on murine KLN205 NSCLC cells (A) and murine B16-F10 melanoma cells (B) after transfection with human PD-L1. Cells were stained with hPD-L1 or an isotype control IgG antibody. [file Image_2.tif]

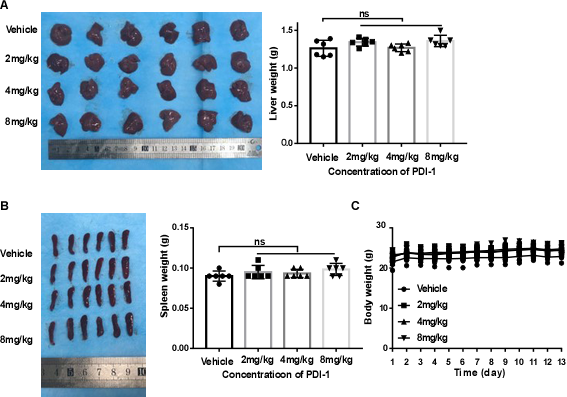

Supplement: Supplementary Figure 3 — PDI-1 exhibits no toxicity in wild-type C57BL/6 mice. (A–C) Groups of 5–6-week-old wild-type C57BL/6 mice were administered vehicle or the indicated doses of PDI-1 by intraperitoneal injection once daily for 13 days. Liver (A), spleen (B) and body (C) weights were determined on day 13. Data represent the mean ± SEM of 6 mice/group. ns, not significant. [file Image_3.tif]

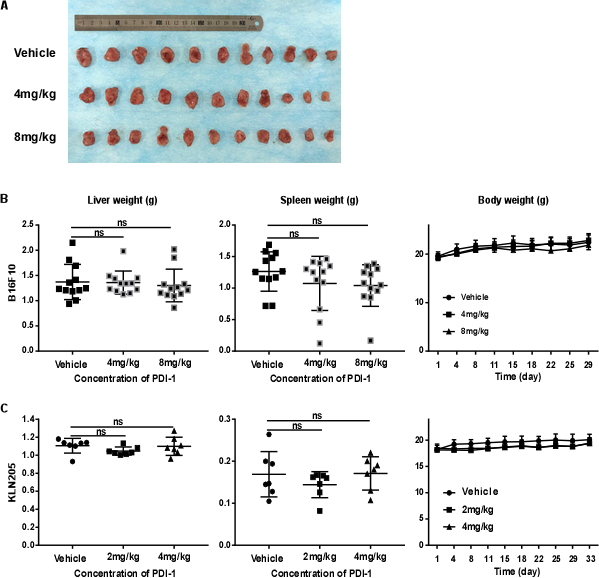

Supplement: Supplementary Figure 4 — PDI-1 exhibits no toxicity in mice bearing hPD-L1-transfected melanoma or NSCLC tumors. (A, B) Groups of C57BL/6 mice (n=11) were injected with hPD-L1-B16-F10 melanoma cells and administered vehicle or the indicated doses of PDI-1 by intraperitoneal injection once daily for 29 days. Tumors were excised and photographed (A) and the liver, spleen, and body weights (B) were then determined. (C) Groups of DBA/2 mice (n=8) were injected with hPD-L1-KLN205 NSCLC cells and administered vehicle or the indicated doses of PDI-1 by intraperitoneal injection once daily for 33 days. Liver, spleen, and body weights were then determined. Data represent the mean ± SEM. ns, not significant. [file Image_4.tif]

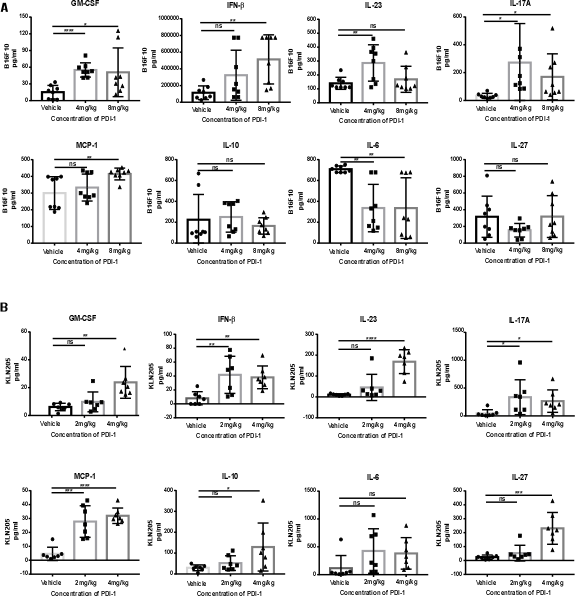

Supplement: Supplementary Figure 5 — PDI-1 increases inflammatory cytokine production in hPD-L1-transfected tumor-bearing mice. (A, B) Blood samples were collected from C57BL/6 mice bearing hPD-L1-B6F10 tumors on day 29 (A) or from DBA/2 mice bearing hPD-L1-KLN205 tumors on day 33 (B). Sera were analyzed for the indicated inflammatory mediators using a multiplex flow cytometry assay. Data represent the mean ± SEM of 8 mice/group. *p < 0.05; **p < 0.01; ***p < 0.001; ****p < 0.0001; ns, not significant. [file Image_5.tif]

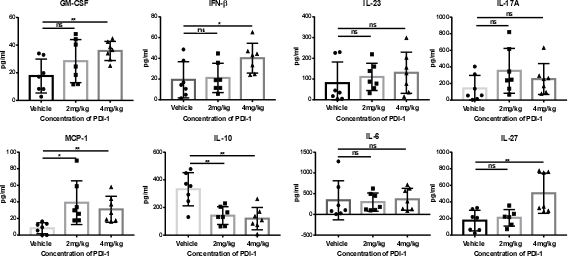

Supplement: Supplementary Figure 6 — PDI-1 rapidly boosts the host immune response to hPD-L1-bearing tumor cells. DBA/2 mice harboring hPD-L1-KLN205 tumors were bled on day 7 and sera were analyzed for the indicated inflammatory mediators using a multiplex flow cytometry assay. Data represent the mean ± SEM of 7 mice/group. *p < 0.05; **p < 0.01; ns, not significant. [file Image_6.tif]

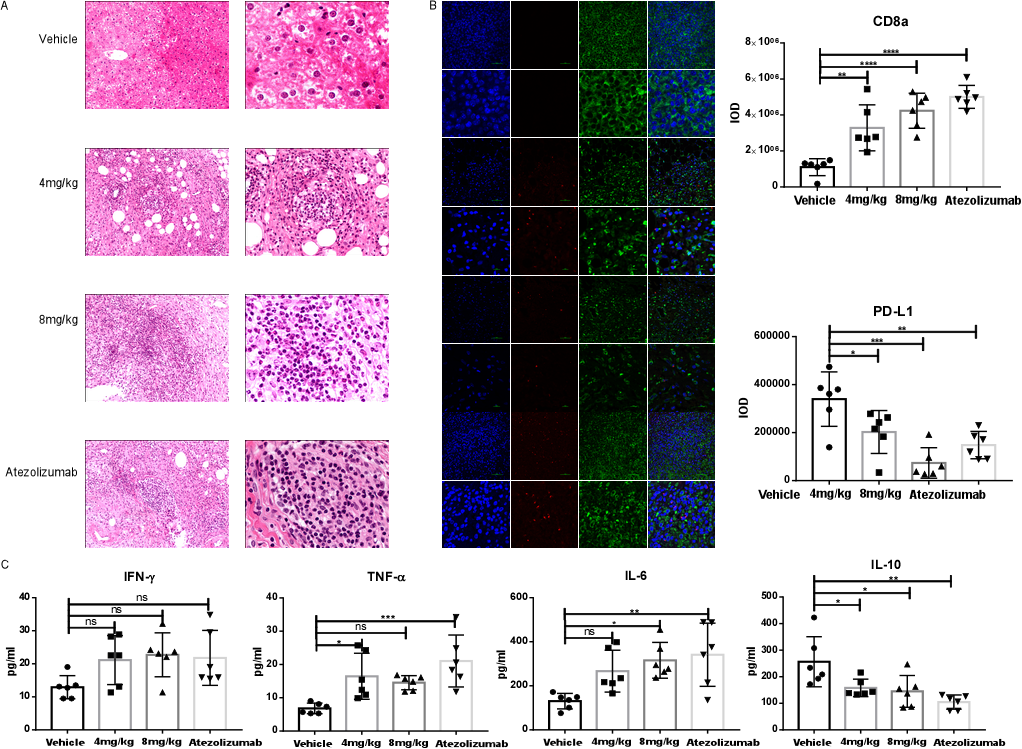

Supplement: Supplementary Figure 7 — Comparison of PDI-1 with Atezolizumab in an animal model. (A–C) Groups of C57BL/6 (n=6) mice were injected with hPD-L1-B16-F10 murine melanoma cells and administered vehicle, 4 or 8 mg/kg PDI-1, or the anti-PD-L1 mAb atezolizumab (10 mg/kg) by intraperitoneal injection once daily for 7 days. Tumors and blood samples were collected on day 14. (A) Representative images of H&E-stained tumor sections. (B) Fluorescent multiplex immunohistochemical staining of PD-L1, CD8a, and FoxP3 protein in excised tumors. (C) Serum levels of the indicated cytokines were determined using a multiplex flow cytometry assay. *p < 0.05; **p < 0.01; ***p < 0.001; ****p < 0.0001; ns, not significant. [file Image_7.tif]

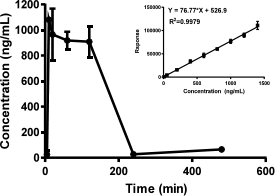

Supplement: Supplementary Figure 8 — Distribution of PDI-1 in mice. MRM chromatograms for PDI-1 detected in the serum of C57BL/C male mice. [file Image_8.tif]
